# Combined Treatment with an Anticoagulant and a Vasodilator Prevents Steroid-Associated Osteonecrosis of Rabbit Femoral Heads by Improving Hypercoagulability

**DOI:** 10.1155/2017/1624074

**Published:** 2017-10-19

**Authors:** Fang Cao, Ge Liu, Wei Wang, Benjie Wang, Xiaowei Wei, Faqiang Lu, Fan Yang, Kai Kang, Yongxuan Wang, Jiahui Yang, Kairong Qin, Dewei Zhao

**Affiliations:** ^1^Department of Biomedical Engineering, Faculty of Electronic Information and Electronical Engineering, Dalian University of Technology, Dalian 116024, China; ^2^Department of Orthopaedic Laboratory, Affiliated Zhongshan Hospital of Dalian University, Dalian 116001, China

## Abstract

Steroid-associated osteonecrosis of the femoral head remains a challenging problem in orthopedics worldwide. One pathomechanism is ischemia of the femoral head, as a result of thrombus formation and vasoconstriction. The present study investigates the effects of combination prevention with enoxaparin and EGb 761 on steroid-associated ONFH in rabbits. Rabbits were randomly divided into 5 groups (control group, model group, enoxaparin group, ginkgo group, and combination group). With the exception of the control group, the groups of rabbits were modeled with lipopolysaccharide and methylprednisolone acetate. Starting with modeling, the enoxaparin group and ginkgo group were injected with 1 *μ*g/kg/day enoxaparin subcutaneously and orally given 40 mg/kg/day EGb 761 for 4 weeks, respectively; the combination group received both treatments. After modeling for 6 weeks, the hematology data indicated prolonged PT and APTT in the three prevention groups. The micro-CT examination revealed higher bone density and better structure; histomorphometry revealed significant pathological changes. Immunohistochemistry revealed higher expression of BMP-2 and VEGF, thus revealing better osteogenesis and angiogenesis activities. Among the three prevention groups, the combination group had the most efficient results. In conclusion, the combined prevention with an anticoagulant and a vasodilator has the potential to decrease the incidence of steroid-associated ONFH in rabbits.

## 1. Introduction

Osteonecrosis of the femoral head (ONFH) has been reported to occur in patients who undergo long-term or heavy glucocorticoid use as a treatment for underlying diseases, such as systemic lupus erythematosus, nephrotic syndrome, and renal transplantation [1]. ONFH, as a progressive and degenerative bone disease, leads to the collapse of the femoral head, which subsequently destroys the hip joint and affects the patient's activities [2]. One of the most common treatment options for patients with advanced ONFH is total hip replacement. However, steroid-associated ONFH tends to occur in individuals aged 30–50 y, and the joint prosthesis is not sufficiently durable for younger patients with steroid-associated ONFH [3, 4]. Effective nonsurgical treatment in the early stages of ONFH would be preferable to total hip replacement.

The precise pathogenesis of steroid-associated ONFH remains unclear. Several possible factors relating to the pathogenesis of ONFH have been suggested on the basis of both human and animal studies, including coagulation abnormalities, hyperlipidemia, endothelial dysfunction, and oxidative stress [5–9]. These factors have been thought to be related to an interruption of the bone vascular circulation and to result in bone ischemia. Moreover, many experimental studies have revealed that glucocorticoids directly injure endothelial cells, thus resulting in vasoconstriction, disturbance of the coagulation-fibrinolysis system, and thrombus formation in the femoral head, which consequently decrease the blood circulation of the trabecular bone and finally lead to ONFH [10, 11]. Therefore, maintaining the blood circulation of the femoral head is likely to be an effective measure to prevent steroid-associated ONFH.

An insufficient blood circulation to the femoral head is likely caused by thrombus formation and vasoconstriction [12, 13]. In the present study, enoxaparin, a low molecular weight heparin, was used as an anticoagulant to prevent thrombosis. Glueck et al., in a human pilot study, have demonstrated that enoxaparin prevents the progression of Ficat stages I and II osteonecrosis of the hip associated with thrombophilia or hypofibrinolysis or both [14]. In a rat model of mechanical induced osteonecrosis generated by incising the periosteum of the neck and cutting the ligamentum teres, enoxaparin has an anticoagulant effect preventing the occurrence of osteonecrosis [15]. Enoxaparin treatment, when simultaneously administered with high dose glucocorticoid in rabbits, decreases empty lacunae and necrotic osteocytes [12]. Extracts of* Ginkgo biloba *leaves (EGb 761) have effects including dilating blood vessels, preventing thrombus formation, decreasing levels of blood lipids, scavenging free radicals, and preventing lipid peroxidation [16]. It has been widely used in the prevention and treatment of cardiovascular and peripheral blood vessel diseases [17, 18]. EGb 761 induces the relaxation of the rat thoracic aorta and is associated with an increase in endothelial intracellular calcium levels [19]. In in vivo experiments after induction with a high cholesterol diet (HCD), EGb 761 improved endothelial function by lowering total plasma cholesterol levels in hamsters [20].

The effects of either an anticoagulant or a vasodilator alone to prevent glucocorticoid-associated ONFH have recently been assessed. In this study, the model of glucocorticoid-associated ONFH was established in rabbits and we used combined treatment with an anticoagulant (enoxaparin) and a vasodilator (EGb 761), which prevented the development of glucocorticoid-associated ONFH more effectively than either treatment alone in rabbits. We also evaluated how enoxaparin and EGb 761 influenced hematological parameters.

## 2. Materials and Methods

### 2.1. Animals

Adult male healthy New Zealand white rabbits weighing 2.5–3.5 kg were purchased from Dalian Medical University in Dalian, China. All of the procedures were conducted according to the care and use of laboratory animals guidelines established by the National Institutes of Health; rabbits were housed in conventional cages and a standard laboratory diet and water were maintained. Before the experiment, the rabbits were acclimated for 1 week.

### 2.2. Drugs and Chemicals

Enoxaparin (Clexane, Sanofi, France) and EGb 761 (Ginaton, Germany) were purchased from the Affiliated Zhongshan Hospital of Dalian University (Dalian, China).

The EGb 761 used in our study was standardized to 9.6 mg Ginkgo flavone glycosides and 2.4 mg terpene lactones (Ginkgolides, Bilobalide).

### 2.3. Experimental Design

Rabbits were divided into 5 groups and were processed as follows. Control group (*n* = 6): it was the normal group; rabbits were given equal volumes of saline. Model group (*n* = 6): rabbits were intravenously injected with 10 *μ*g/kg lipopolysaccharide (Sigma, USA) and then injected intramuscularly with 20 mg/kg of methylprednisolone acetate (Pfizer, USA) 3 times with a time interval of 24 h to construct the model. The steroid-associated ONFH rabbit model was prepared using a previously published inductive protocol [21]. Enoxaparin group (*n* = 6): model rabbits received only enoxaparin (subcutaneous injection, 1 *μ*g/kg/day) for 4 weeks. Ginkgo group (*n* = 6): model rabbits received only EGb 761 (oral administration, 40 mg/kg/day) for 4 weeks. Combination group (*n* = 6): model rabbits received enoxaparin (subcutaneous injection, 1 *μ*g/kg/day) combined with EGb 761 (oral administration, 40 mg/kg/day) for 4 weeks. Six weeks later, the rabbits were sacrificed, and the femoral heads of the rabbits in each group were obtained and assessed with micro-CT scanning and histopathology and immunohistochemistry analyses.

### 2.4. Coagulation Measurement

Blood samples were collected from the marginal ear veins while the animals were in a fasting state. The samples were obtained in the early morning at week 6 after the methylprednisolone injection. The platelet free plasma was used to determine the prothrombin time (PT) and the activated partial thromboplastin time (APTT).

### 2.5. Micro-CT Assay

All femoral head samples were scanned with an Inveon Micro-CT manufactured by Siemens (Berlin, Germany) with mid-high resolution. The scanning protocol was 80 kV and 500 *μ*A, with an effective pixel size of 15.48 *μ*m. A volume of interest (VOI) was selected from these regions for three-dimensional reconstruction, and the following bone parameters were analyzed: CT value, bone volume/total volume (BV/TV), trabecular thickness (Tb.Th, mm), and trabecular spacing (Tb.Sp, mm).

### 2.6. Tissue Sample Preparation

The rabbits were euthanized at 6 weeks. The femoral heads from both sides were harvested. The femoral heads were fixed in 4% neutral paraformaldehyde solution for 7 days and decalcified in 10% ethylene diamine tetraacetic acid (EDTA) buffer (pH = 7.2). The EDTA buffer was replaced every 7 days. The degree of decalcification was measured using micro-CT. The femoral head was gradually dehydrated using dimethyl benzene and embedded in paraffin after 8 weeks of decalcification. The paraffin block was cut into 4-mm-thick sections and glued onto microslides pretreated with polylysine. The sections were stained with hematoxylin and eosin (H&E).

### 2.7. Evaluation of Steroid-Associated ONFH

The osteonecrotic changes and repair processes in steroid-treated rabbits were observed by histopathological examination using a light microscope six weeks after modeling. The slides were evaluated in a blinded fashion by three independent observers. The evaluation criteria for osteonecrosis were based on a report by Yamamoto et al. [22]. Osteonecrosis was deemed to be present when there was necrosis of medullary hematopoietic cells or fat cells or there were empty lacunae or condensed nuclei in osteocytes. The number of empty lacunae in five random regions in each section (5 sections in each group) was counted and the percentage of empty lacunae was calculated.

### 2.8. Immunohistochemistry

To analyze the angiogenesis and osteogenic potential in the femoral head, immunohistochemistry was performed using antibodies specific for vascular endothelial growth factor (VEGF) (mouse antibody, dilution 1 : 200, Abcam, Cambridge, MA, UK) and bone morphogenetic protein-2 (BMP-2) (mouse antibody, dilution 1 : 100, Abcam, Cambridge, MA, UK). Briefly, sections were immersed in 3% hydrogen peroxide in 0.01 M phosphate-buffered saline (PBS) for 10 min to block endogenous peroxidase activity and then were rinsed several times in PBS. After being blocked with 10% goat normal antiserum (Solarbio, Beijing, China) for 30 min at room temperature, sections were treated with a primary antibody overnight at 4°C and incubated with the HRP-conjugated secondary antibody for 30 min. To reveal the immunoreactivity (IR), the sections were then reacted with diaminobenzidine solution in the dark. Finally, the sections were treated with hematoxylin and mounted. The sections without primary antibody processing were used as negative controls. Each test was repeated 3 times. The sections were observed and photographed. ImagePro Plus 6.0 software was used to analyze quantitatively and to calculate the mean density. Five random regions in each section (3 sections in each group) were selected and the mean density was calculated as the ratio of IOD to the selected area.

### 2.9. Statistical Analysis

Data were analyzed using GraphPad Prism 6.0 statistical software and are expressed as the means ± SEM. A one-way ANOVA was used to analyze the significant differences among the different groups. Comparisons between two groups were performed with Tukey tests. *p* < 0.05 was considered to indicate a statistically significant difference between groups.

## 3. Results

### 3.1. Coagulation Analysis

**Figure 1:**
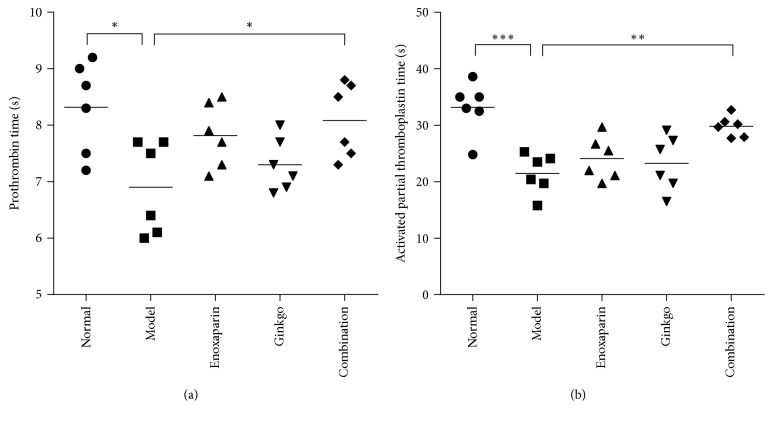
*Coagulation analysis*. Hematological data of PT (a) and APTT (b) in the five groups of rabbits (normal group (●), model group (■), enoxaparin group (▲), ginkgo group (▼), and combination group (◆)). Data are expressed as the mean (*n* = 6); ^*∗*^*p* < 0.05; ^*∗∗*^*p* < 0.01; ^*∗∗∗*^*p* < 0.001.

To investigate the coagulation state of the model group and the potential effects of enoxaparin and EGb 761, the coagulation states were evaluated by measuring the PT and APTT. In the model group, PT was 6.90 ± 0.33 s, significantly lower than that of the control group (8.32 ± 0.33 s, *p* = 0.0106 < 0.05), which showed hypercoagulability of the femoral heads. Compared with model group, enoxaparin group and ginkgo group resulted in a prolongation of PT but not significant, which was 7.82 ± 0.23 s (*p* = 0.1662) and 7.30 ± 0.19 s (*p* = 0.8436), respectively; the combination group showed the best results: the PT reached 8.08 ± 0.27 s, representing a significant prolongation compared with model group (*p* = 0.0418 < 0.05) (Figure 1(a)). The effect on APTT is shown in Figure 1(b). Neither enoxaparin nor ginkgo caused significant prolongation in APTT, which was 21.47 ± 1.44 s (*p* = 0.7625) and 24.12 ± 1.56 s (*p* = 0.9321), respectively, compared with that in the model group (20.15 ± 1.89 s). The APTT of the combination group was 29.80 ± 0.76 s (*p* = 0.0084 < 0.01), significantly lower than the model group, and was closest to the control group (33.15 ± 1.89 s).

### 3.2. Micro-CT

**Figure 2:**
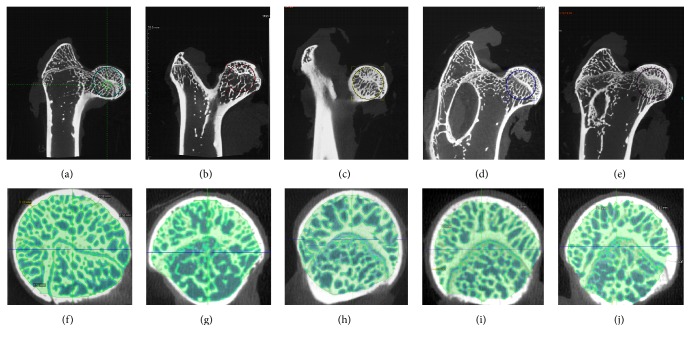
*Micro-CT scanning images*. A representative micro-CT image from the model group (b, g) showed that the trabecular bone was sparser in the subchondral bone compared with the control group (a, f). In the prevention groups (enoxaparin group (c, h), ginkgo group (d, i), and combination group (e, j)), the trabecular bone was thicker and denser. The circles in the images represented the chosen area to analyze the micro-CT parameters.

**Figure 3:**
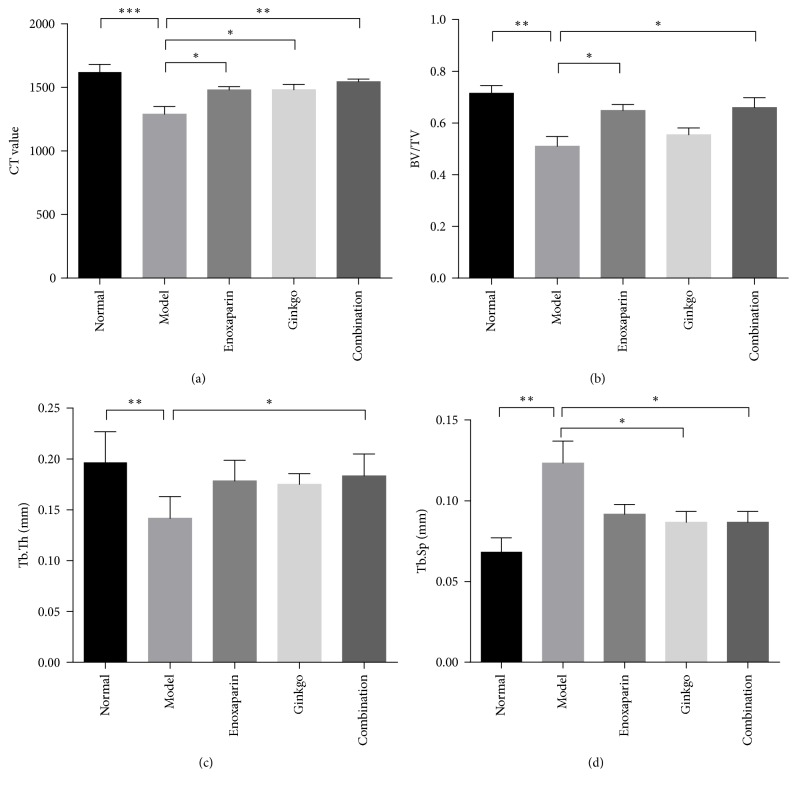
*Micro-CT data*. Parameters analysis of the micro-CT was performed on the differences in CT values (a), BV/TV (b), Tb.Th (c), and Tb.Sp (d) in the 5 groups. Data are presented as the mean ± SD (*n* = 6). ^*∗*^*p* < 0.05; ^*∗∗*^*p* < 0.01; ^*∗∗∗*^*p* < 0.001.

Micro-CT images indicated that the trabecular bone was sparser and more disordered in the subchondral bone in the model group compared with the control group (Figure 2). The CT value, mean rate value of bone volume and total volume (BV/TV), and trabecular thickness (Tb.Th) in the model group were all lower than those in the control group, whereas the trabecular spacing (Tb.Sp) was higher. The data from the trabecular bone showed changes in the three prevention groups. The CT value in the combination group (1546 ± 20.50) was significantly higher (*p* < 0.05) than that in the model group (1291 ± 59.46). The BT/TV in the combination group (0.66 ± 0.09) was significantly higher (*p* < 0.05) than that in the model group (0.51 ± 0.04). The Tb.Th was greater (*p* < 0.05) in the combination group (0.18 ± 0.01 mm) than in the model group (0.14 ± 0.02 mm). The Tb.Sp was lower (*p* < 0.05) in the combination group (0.087 ± 0.007 mm) than in the model group (0.12 ± 0.013 mm) (Figure 3).

### 3.3. Histology

**Figure 4:**
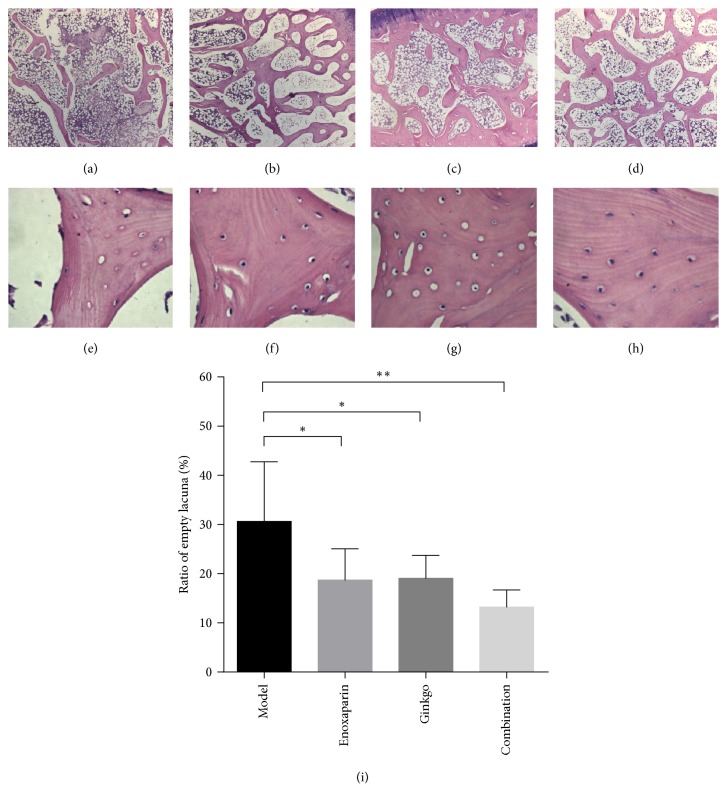
*Histological observation.* Representative photomicrographs of the subchondral bone (a, b, c, d), the empty lacunae (e, f, g, h), and ratio of empty lacunae (i) of the femoral heads in the four groups. (a, e) The model group showed sparser and more disordered trabecular bone with more empty lacunae, surrounded by fewer marrow cells and more marrow fat cells. In the (b, f) enoxaparin group and (c, g) the ginkgo group, the trabecular bone ranged more regularly. A decreased number of empty lacunae were observed. (d, h) The combination group showed fewer empty lacunae, and the trabecular bone was surrounded with normal marrow. Stain: hematoxylin and eosin; magnification: ×40 (a, b, c, d), ×200 (e, f, g, h). Data are presented as the mean ± SD. ^*∗*^*p* < 0.05; ^*∗∗*^*p* < 0.01.

Histological examinations revealed that the typical early stage of osteonecrosis was observed in the model group. The trabecular bone displayed a disordered structure, which appeared thinner and sparser, the fracture of trabecular bone led to the formation of necrotic bone tissue, and the trabecular space was larger. In addition, osteoclasts were observed, and the apoptosis of osteocytes led to the appearance of more empty lacunae (30.63 ± 12.11%) accompanied by local bone marrow cell necrosis or myelofibrosis; hypertrophic fat cells and the adipose area in bone marrow were increased. In the enoxaparin and ginkgo groups, compared with the model group, the trabecular bone ranged regularly, and fewer empty lacunae were observed, 18.72 ± 6.23% (*p* = 0.038 < 0.05) and 19.08 ± 4.63% (*p* = 0.046 < 0.05), respectively. In the enoxaparin and ginkgo groups, hematopoietic cells in the bone marrow cavity were rich, and osteoclasts were not found. There were no obvious pathological changes appearing in the combination group, and the ratio of empty lacunae is 13.22 ± 3.45 (*p* = 0.0012 < 0.01) compared with the model group (Figure 4).

### 3.4. Immunohistochemistry

**Figure 5:**
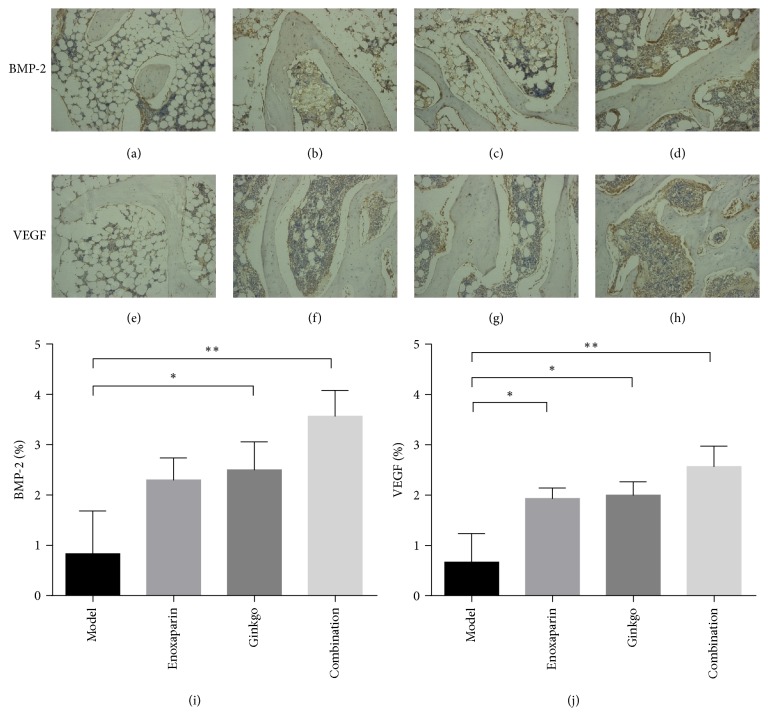
*Immunohistochemical staining*. Analysis of BMP-2 and VEGF protein in the femoral heads from each group (100x magnification). The model group (a, e) showed slight BMP-2 immunoreactivity and VEGF immunoreactivity in the bone tissue. In the enoxaparin group (b, f) and ginkgo group (c, g), increased BMP-2 and VEGF expressions were observed. The combination group (d, h) showed intense expression of BMP-2 and VEGF compared with that in the other groups, especially in the bone marrow. Quantitative analysis of BMP-2 (i) and VEGF (j) protein was performed in each group and the mean density was calculated. Data are presented as the mean ± SD. ^*∗*^*p* < 0.05; ^*∗∗*^*p* < 0.01.

The expression of angiogenic factors including VEGF and osteogenic factors including BMP-2 in the femoral heads from each group was analyzed by immunohistochemical staining and quantitative analysis. The appearance of a yellow brown substance indicated positive staining (a, e). The model group showed slight BMP-2 (Figures 5(a) and 5(i)) immunoreactivity and VEGF (Figures 5(b) and 5(j)) immunoreactivity in the bone tissue. In the enoxaparin group and ginkgo group, increased BMP-2 (Figures 5(b), 5(c), and 5(i)) and VEGF (Figures 5(f), 5(g), and 5(j)) expression levels were observed. The combination group showed significant increased expressions of BMP-2 (Figures 5(d) and 5(i)) and VEGF (Figures 5(h) and 5(j)) compared with the model group, especially in the bone marrow.

## 4. Discussion

In this study, we successfully constructed a model of early stage steroid-associated ONFH in rabbits, which was based on the osteonecrosis model established by Qin et al. [21]. Only early stage changes of ONFH appeared; there was no subchondral bone collapse demonstrated in this model or pathogenetic changes of steroid-associated ONFH in rabbits similar to those observed in human ONFH. But rabbits do not walk upright, so the model can not completely simulate the load bearing of human femoral head. We used this model to detect whether the combined applications of enoxaparin and EGb 761 could be used as a preventive therapy against steroid-associated ONFH. Glucocorticoids led to osteonecrosis in the femoral head, as demonstrated by the increased number of empty lacunae and necrotic osteocytes. The simultaneous administration of enoxaparin and EGb 761 in the osteonecrosis model prolonged blood coagulation time and decreased the number of necrotic osteocytes and empty osteocyte lacunae, as compared with the model group. This result suggested that enoxaparin and EGb 761 prevent steroid-associated ONFH effects on circulation and bone by improving hypercoagulability.

Steroid-associated ONFH is closely related to hypercoagulability. Reports have suggested that intravascular coagulation (IC), as an intermediary mechanism caused by hypercoagulability and intraosseous fat embolism, is the most common pathway leading to nontraumatic ON [23]. The altered fat metabolism could increase serum lipid levels (triglyceride and cholesterol) and a fat embolism resulted in vascular occlusion which accelerated the ischemia. It is reported that the inhibition of both thrombosis and lipid deposition could reduce the incidence of steroid-associated ONFH. Hematologic evidence of intraosseous hypercoagulability has been found on the basis of both human and animal studies of ON [24, 25]. Hypercoagulability, a pathological process mediated by a variety of factors and hematological changes, plays an important role in the process of thrombus formation [26]. Moreover, steroid treatment enhances hypercoagulability, induces venous thrombosis, decreases blood circulation, and leads to ONFH [27, 28]. Consistent with this idea, blood flow reduction in the femoral head and hypercoagulability of plasma have been observed in a swine model after 24 h of steroid treatment [29]. Likewise, high dose steroid administration in a rabbit model has been found to potentiate the magnitude of osteonecrosis by increasing hypercoagulability, which subsequently induces thrombus formation [30]. Additionally, steroids are known to modulate other vasoactive mediators. They facilitate the expression of vasoconstrictors such as endothelin-1 [31] and suppress the production of vasodilators such as bradykinin, prostacyclin, and nitric oxide [32–34]. Hence, steroids enhance the constriction of femoral head arteries by modulating vascular responsiveness to vasoactive substances. Vasoconstriction causes a decrease in femoral head blood flow and exacerbates the hypercoagulability in the femoral head [9]. Hypercoagulability or vasoconstriction induced by steroids contributes to femoral head ischemia, thus ultimately leading to ONFH. Therefore, maintaining the blood circulation in the femoral head is necessary to prevent steroid-associated ONFH.

Our study demonstrated administration of high dose steroids-associated ONFH in rabbits. We observed signs of early osteonecrosis in the model group. The micro-CT result showed that the CT value, BV/TV, and Tb.Th decreased significantly, whereas the Tb.Sp increased significantly in the model group. Moreover, histopathological examination also showed that the trabecular structure was disordered and that the number of empty lacunae was increased in the model group. At the same time, we also detected coagulation parameters, PT and APTT. The PT (*p* < 0.05) and APTT (*p* < 0.001) in the model group were significantly lower than those in the control group at 6 weeks after steroid administration. PT and APTT are performance indicators reflecting the efficiency of both the intrinsic and extrinsic coagulation pathways. The shortening of PT and APTT reflects a hypercoagulable blood state and a tendency toward thrombosis. Therefore, our study showed that steroids cause hypercoagulability, thereby promoting thrombosis and femoral head ischemia, and finally leading to ONFH. These results are consistent with findings from previous studies [30].

Drops of* Ginkgo biloba* leaf extract have many pharmacological effects. The extract promotes a maintenance of arterial and venous vascular tone via stimulation of catecholamine release and inhibition of degradation, together with an arterial relaxant effect via stimulation of prostacyclin and endothelium-derived relaxing factor production [35]. This extract has widely been used in the prevention and treatment of cardiovascular and cerebrovascular diseases and peripheral vascular diseases [17, 18, 36]. However, the use of this extract in the prevention of ONFH has rarely been reported [37]. It not only leads to the vasodilation of blood vessels but also promotes osteogenesis [20]. Oral administration of EGb 761 has been found to restore bone mineral density and microstructure in aged ovariectomized rats [38]. EGb 761 has been found to promote osteoblast genesis and to decrease bone marrow adipogenesis [39]. On the basis of the effects of the extract of* Ginkgo biloba* leaf drops, we chose EGb 761 as a vasodilator to relieve steroid-associated vasoconstriction. In addition, enoxaparin was chosen as an anticoagulant to prevent steroid-associated thrombosis, on the basis of previous clinical studies [14] and animal studies [12]. The results of this study showed that PT and APTT were prolonged slightly more in the enoxaparin group and ginkgo group, but there were no significant differences compared with the model group. When we simultaneously administered enoxaparin and EGb 761 to the high dose steroid group, PT (*p* < 0.05) and APTT (*p* < 0.01) showed a significant prolongation, as compared with the model group. Bone repair was evaluated by micro-CT scanning and HE staining. Micro-CT scanning indicated that the combination group had more new bone formation and better microstructural parameters than the model group, a result further confirmed by HE staining. The results of this study preliminarily showed that the combined effects of enoxaparin and EGb 761 prevent steroid-associated ONFH by improving hypercoagulability in rabbits.

The local expression levels of BMP-2 and VEGF were detected in this study. BMP-2 has an important and significant role in inducing osteogenesis, the rehabilitation and reconstruction of the femoral head in response to necrosis. Studies have demonstrated that the content of BMP-2 reflects the bone formation ability of osteoblasts [40]. The main functions of VEGF are to promote the regeneration of blood vessels by mitosis of vascular endothelial cells and to increase vascular permeability [41]. In addition, VEGF has been reported to promote bone repair and bone regeneration in part by inducing angiogenesis [42]. Glucocorticoid decreases the expression of BMP-2, thereby inhibiting the maturity of osteoblasts and slowing the process of bone formation [43]. Meanwhile, it decreases the expression of VEGF and disrupts the process of vascularization and the growth of new blood vessels in the necrotic bone [44]. In our study, immunohistochemical results showed that the expression of BMP-2 and VEGF in the femoral heads significantly increased in the combination group. This finding demonstrated that the combined applications of enoxaparin and ginkgo enhanced angiogenesis and maintained bone structure by improving hypercoagulability, which in turn prevented the occurrence of steroid-associated ONFH in rabbits.

Thus, these results showed that the combined application of an anticoagulant (enoxaparin) and a vasodilator (EGb 761) can prevent or delay the progression of the early stages of steroid-associated ONFH. Although this treatment cannot completely stop the progression of steroid-associated osteonecrosis, it appears to enhance angiogenesis-osteogenesis in femoral heads. Although no bleeding site was found in the experiment, possible systemic side effect of enoxaparin or ginkgo may not have been controlled well and may thus have resulted in a risk for bleeding. Thus, more tests should be made to verify and evaluate the risk of bleeding before this preventive therapy could apply clinically in human steroid-associated osteonecrosis of femoral head. In addition, precise molecular mechanisms of this prevention method should be clarified in the future.

In summary, this study indicated that the combined administration of enoxaparin and EGb 761 enhances angiogenesis and bone repair by improving hypercoagulability associated with early stage ONFH in a rabbit model and may provide an efficient, inexpensive, and simple therapy to prevent early stage ONFH.
